# Influence of Controlled Epoxidation of an Asymmetric Styrene/Butadiene Star Block Copolymer on Structural and Mechanical Properties

**DOI:** 10.3390/polym13010096

**Published:** 2020-12-29

**Authors:** Shankar P. Khatiwada, Ulrike Staudinger, Dieter Jehnichen, Gert Heinrich, Rameshwar Adhikari

**Affiliations:** 1 Research Center for Applied Science and Technology (RECAST), Tribhuvan University, P. O. Box 1030, Kirtipur, Kathmandu 44613, Nepal; skradius33@gmail.com (S.P.K.); nepalpolymer@yahoo.com (R.A.); 2 Nepal Polymer Institute (NPI), P. O. Box 24411, Kathmandu 44613, Nepal; 3 Leibniz-Institut für Polymerforschung Dresden e.V., Hohe Str. 6, 01069 Dresden, Germany; djeh@ipfdd.de (D.J.); gheinrich@ipfdd.de (G.H.); 4 Institut für Textilmaschinen und Textile Hochleistungswerkstofftechnik, Technische Universität Dresden, 01069 Dresden, Germany

**Keywords:** star block copolymer, epoxidation, morphology, mechanical properties, nanostructures

## Abstract

The chemical modification (namely the epoxidation) of a star shaped block copolymer (BCP) based on polystyrene (PS) and polybutadiene (PB) and its effect on structural and mechanical properties of the polymer were investigated. Epoxidation degrees of 37 mol%, 58 mol%, and 82 mol% were achieved by the reaction of the copolymer with *meta*-chloroperoxy benzoic acid (*m*-CPBA) under controlled conditions. The BCP structure was found to change from lamellae-like to mixed-type morphologies for intermediate epoxidation level while leading to quite ordered cylindrical structures for the higher level of chemical modification. As a consequence, the glass transition temperature (*T_g_*) of the soft PB component of the BCP shifted towards significantly higher temperature. A clear increase in tensile modulus and tensile strength with a moderate decrease in elongation at break was observed. The epoxidized BCPs are suitable as reactive templates for the fabrication of nanostructured thermosetting resins.

## 1. Introduction

Styrene/butadiene (SB) block copolymers (BCPs) represent a group of advanced materials with a wide range of applications [1,2], in which the polymers synergistically combine the hard and rigid thermoplastic nature of polystyrene (PS) with the soft and flexible segments of polybutadiene (PB). Due to the covalent bonds between the two components, microphase-separated morphologies in the nanometer range are formed, resulting in the transparency of these materials [3,4,5,6,7]. Changing the molecular architecture, relative chemical composition, and nature of monomers the properties of the BCPs can be adjusted over a wide range covering a material portfolio from thermoplastic elastomers [8,9] to toughened thermoplastics [10,11,12,13,14,15]. In addition to their use as components in medical equipment, household technology, and automotive industry or the information technology (IT) sector, SB-based BCPs are also used, for example, in adhesives and sealants and asphalt formulations [16,17,18,19]. In addition, such BCPs have become interesting candidates for use as templates for the nanostructuring of epoxy resins to increase their impact strength [20,21,22]. However, because of the immiscibility of the BCSs with epoxy resin, blending would generally lead to macrophase separated structures. Thus, in order to enhance their compatibility with the epoxy system, the BCPs can be chemically modified by epoxidation of the PB block. One of the common strategies to epoxidize diene group containing polymers involves the in situ formation of peracids through the reaction between formic acid and hydrogen peroxide [23,24,25,26,27]. However, this type of epoxidation process was reported to go in parallel with several side reactions degrading the BCPs, which can be attributed to the high temperature required for attain high epoxidation degrees. Additionally, above 50 mol% of epoxidation, the epoxide groups were found to undergo a ring opening reaction forming esters and alcohols [24]. Therefore, attempts have been made to epoxidize SB-based BCPs containing olefinic double bonds using *meta*-chloroperoxy benzoic acid (*m*-CPBA) [28,29]. The chemical modification of hydroxyl terminated polybutadiene (HTPB) by *m*-CPBA and later structural clarification of modified HTPB was first reported by Aguiar et al. [30]. Saffer and Johnson originally used *m*-CPBA as an oxidant for qualitatively determination of the double bonds (C=C) in PB [31]. The epoxidation of an SB star block copolymer using the method of Antonietti et al. [28] was reported by Serrano et al. [32]. Due to a large number of undesired reactions accompanying the synthesis, such processes yielded insoluble products having high molar mass. Hence, hydrogen peroxide containing a biphasic water/dichloroethane media was used for epoxidation reaction.

However, Pandit et al. [33] studied the epoxidation of a SB star block copolymer by using four different methods and declared the use of m-CPBA method as the most suitable in terms of cost, for preparing materials with a qualitatively well-defined epoxidation degree. Further, the suitability of such epoxidized samples as templates for nanostructured epoxy resins has been recently demonstrated [34].

Due to the limited number of literature works available on the epoxidation of SB block copolymers, the present study aims to optimize experimental conditions for precise epoxidation of a star shaped styrene/butadiene BCP using *m*-CPBA. The study attempts to adjust variable epoxidation degrees and to characterize its impact on structural and mechanical behavior of the polymer.

## 2. Experimental

### 2.1. Materials

The SB-based star block copolymer, the Styrolux 3G55, purchased from INEOS Styrolution Group GmbH (Frankfurt am Main, Germany), hereafter referred to as ST, was used in this study. The synthesis of star shaped Styrolux grade copolymers is described elsewhere [35]. In brief, the BCP was synthesized by twofold addition of an initiator and a final coupling step. ST has an overall styrene content of 75 wt.%. The molecular architecture of the BCP may be envisioned as being an asymmetric star having a random poly(styrene-co-butadiene) as core (containing 15 wt% of the PS), one longer and three shorter outer polystyrene arms. The number average molecular weight *M_n_*, (determined by gel permeation chromatography using polystyrene as standard) is 86 kg/mol, with a disparity (*Ð*) of ~2.1 [36].

### 2.2. Epoxidation of BCP with m-CPBA

**Figure 1:**
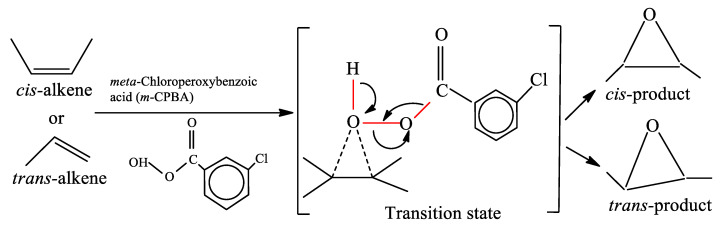
Chemical reaction mechanism of the *meta*-chloroperoxy benzoic acid (*m*-CPBA)/block copolymer (BCP) system.

The epoxidation of the polybutadiene segments of BCP using *m*-CPBA was performed following the standard method as described by Margaritis et al. [29]. For the reaction, 10 g of ST and 200 mL of dichloromethane were taken into a three-necked flask. The mixture was stirred until the content turned into homogenous solution. Varying concentrations of *m*-CPBA were added to the reaction mixture to adjust varying degrees of epoxidation (*DOE*) of 50 mol%, 80 mol%, and 100 mol%. Each solution was then agitated under dry N_2_ gas atmosphere for two hours at 0 °C. After completions of the reaction the polymer solution was carefully filtered and the excess amount of *m*-CPBA was extracted with saturated NaHCO_3_ aqueous solution. The solution was further dried with the help of an Na_2_SO_4_ solution. The solid residue was obtained by evaporating the solvent and drying at room temperature under vacuum. Detailed explanation and a scheme of the reaction mechanism are provided in Section 3.1 (Figure 1).

The *DOE* of the different samples was determined by ^1^H-NMR spectroscopy as described in Section 3.1. The name epST-x is used as acronym for the epoxidized samples where “x” represents the degree of epoxidation in mol%. The samples are denoted as epST-38, epST-57 and epST-82.

### 2.3. Sample Preparation

Samples of ST and epST-x were prepared by solution casting. Then, 10 wt% of each polymer was dissolved in dichloromethane for 5 h at 23 °C and the solution was poured into a Petri disc. Films of about 1 mm thickness were obtained by slow evaporation of the solvent over 72 h and further annealing in a vacuum oven at 100 °C for 48 h.

### 2.4. Molecular Characterization

Qualitative and quantitative analysis of virgin ST and its epoxidized versions of different grades were carried out to prove the epoxidation reaction. The functional groups were identified by FTIR. Infrared spectra of the polymer films were taken by means of a Vertex 80 v spectrometer (Bruker Optik GmbH, Ettlingen, Germany) using ATR mode with spectral width between 4000 and 600 cm^−1^.

The validation of epoxidation reaction and exact degree of epoxidation were analyzed by Proton nuclear magnetic resonance (^1^H-NMR) spectroscopy using the spectrometer Advance III 500 (Bruker Biospin GmbH, Rheinstetten, Germany) at a frequency of 500.13 MHz for ^1^H. The specimens were investigated at 30 °C using deuterated chloroform (CDCl_3_) as spectral reference (*δ*(^1^H) = 7.26 ppm).

A High Performance Liquid Chromatrography (HPLC) device (Agilent Technologies Inc., SantaClara, CA, USA) was used to measure the variation of average molecular weight and the molecular weight distribution for the ST and its epoxidized grades by Gel Permeation Chromatography (GPC) using polystyrene standard, with a PL MIXED-B-LS separation column at a flow rate of 1 mL/min.

### 2.5. Morphological Characterization

The morphology of the block copolymers was evaluated using a LIBRA^®^120 transmission electron microscope (TEM) (Carl Zeiss AG, Oberkochen, Germany) at an acceleration voltage of 120 kV. Sections having a thickness of approximately 60 nm were sliced perpendicular to the film surface at −80 °C using a Leica EM UC6/EM FC6 ultramicrotome (Leica GmbH, Wien, Austria) equipped with a diamond knife. The sections were transferred to a carbon grid and the PB-rich phase of the BCP morphology was selectively stained with osmium tetroxide.

Additionally, small-angle X-ray scattering (SAXS) measurements were performed through 1 mm thick sections of the solution cast films using a multi-range device Ganesha300 XL+ (SAXS LAB ApS, Copenhagen, Denmark) equipped with a 2D-detector Pilatus 300K. Thus, during the measurements, the X-ray beam (SAXS) was aligned vertically and the electron beam (TEM) laterally through the BCP film.

### 2.6. Dynamic Mechanical Analysis (DMA)

Dynamic mechanical analysis (DMA) were performed using an ARES G2 Rheometer (TA Instruments, New Castle, DE, USA) in torsion mode. Test specimens with dimensions of 30 mm × 10 mm were cut from the solution cast films. Standard oscillatory temperature sweeps between −125 °C and 105 °C were carried out applying a frequency of 0.1 rad/s and a strain amplitude of 0.01%. Storage (*G′*) and loss (*G*″) moduli and damping coefficient (*tanδ*) were determined to characterize the impact of the epoxidation grade on the phase behavior of the BCP phases.

### 2.7. Mechanical Characterization

To study the stress–strain behavior of the block copolymers tensile tests were carried out at room temperature at a crosshead speed of 50 mm/min according to DIN EN ISO 527-2. For the measurements, a pneumatic Z010 Zwick universal testing machine (ZwickRoell GmbH & Co. KG, Ulm, Germany equipped with a MultiXtens extensometer was used. For each sample, at least five dog bone shaped specimens of 50 mm × 5 mm × 1 mm in dimensions were tested.

## 3. Results and Discussion

### 3.1. Validation of Epoxidation

The mechanism of the epoxidation of ST block copolymer using *m*-CPBA is shown in Figure 1. The oxygen–oxygen bond in *m*-CPBA is highly reactive because of its weak bond energy (about 33 kcal/mol). During the reaction, the bond between the oxygen and the alkene is formed, while at the same time the O-O bond breaks and the proton is transferred from the OH to the carbonyl oxygen. This stage is very reactive and is called the transition state. The stereochemistry of the *m*-CPBA molecule is always maintained, and thus the *trans*-alkene always gives a *trans*-product and the *cis*-alkene the *cis*-product [37].

**Figure 2:**
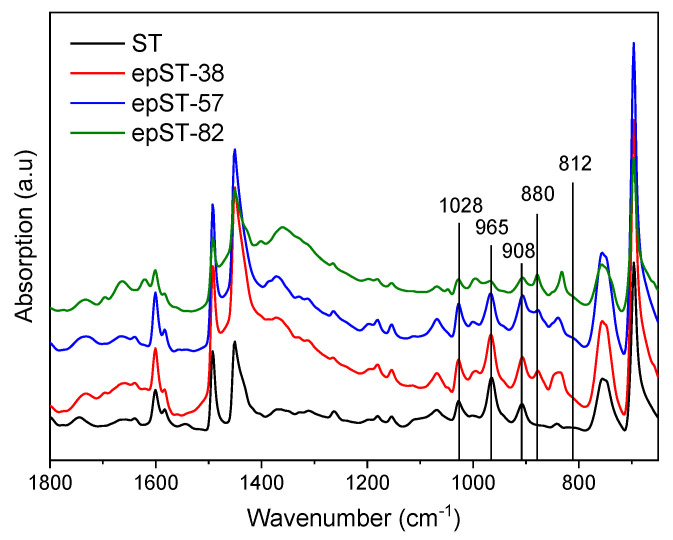
FTIR spectra of virgin Styrolux 3G55 (ST) and its epoxidized versions (epST-x).

The FTIR plots of ST and the epoxidized samples epST-x are shown in Figure 2. For the virgin ST, significant peaks located at about 1028 cm^−1^_,_ 965 cm^−1^, and 908 cm^−1^ were observed. These signals refer to the presence of cis-1,4-polybutadiene groups (-CH_2_-CH=CH-CH_2_-), trans-1,4-polybutadiene groups (-CH_2_-CH=CH-CH_2_-) and the -CH bending vibration of vinyl groups (-CH=CH_2_-), respectively, which has already been observed by Munteanu et al. [38] in an FTIR study on similar SB star block copolymers.

Two additional peaks were identified for all epoxidized grades at 880 cm^−1^ and 812 cm^−1^, which correspond to the addition of oxirane groups at trans- and cis-1,4-butadiene, respectively, and thus confirm successful epoxidation reactions. Additionally, with increasing *DOE*, a decrease of the intensity of the peaks related to cis- and trans-1,4-butadiene was observed. However, the peak corresponding to 1,2-butadiene remained constant indicating that the vinyl butadiene chains were inactive during the reaction, which might be due to its lower affinity towards epoxy groups. Moreover, the lack of additional signals at the 1000–1100 cm^−1^ and 1700–1750 cm^−1^ region in the FTIR spectra indicates the absence of any side reactions, such as ring opening or gelation.

**Figure 3:**
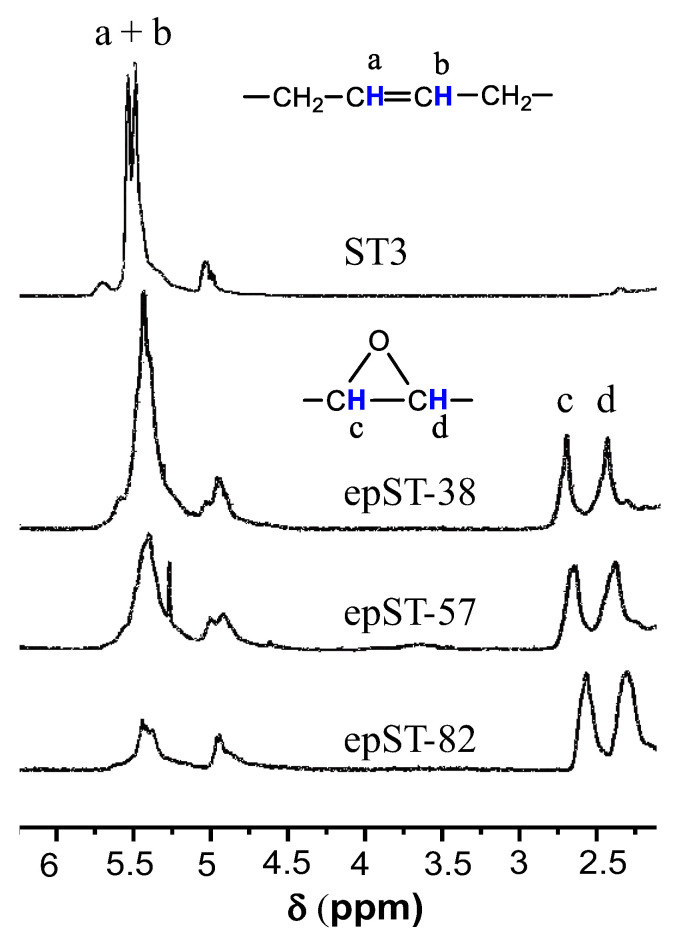
^1^H-NMR spectra of ST and epST-x.

The chemical shifts of the corresponding proton of ST and epST-x observed in the ^1^H-NMR spectra are presented in Figure 3. According to the ^1^H-NMR spectrum, two signals at 5.5 ppm (a) and 5 ppm (b) correspond to the proton of non-epoxidized trans-and cis-1,4-butadiene units, respectively. In comparison, the epoxidized grades have two additional signals at 2.9 ppm (c) and 2.7 ppm (d), which can be assigned to protons, which are attached to cis- and trans-epoxy groups of epoxidized 1,4-butadiene units, respectively.

The integral area of the signals at 5.5 ppm and 5 ppm gradually decreases as the level of epoxidation increases from 38 mol% to 82 mol%. At the same time the integral area of the proton signals at 2.9 ppm and 2.7 ppm progressively increases as the level of epoxidation increases. Furthermore, the signals corresponding to 1,2-butadiene unit of ST do not alter. A chemical modification of vinyl-1,2-butadiene units was not detected by ^1^H-NMR, which is consistent with the results of the FTIR measurement. The ^1^H-NMR and FTIR results of the present study are very much consistent with the observations of Nikje et al., who studied the epoxidation of butadiene and hydroxyl terminated butadiene using dimethyl dioxirane and varying catalysts [39,40].

The ^1^H-NMR spectra were used to calculate the *DOE* of the of the epoxidized samples using the following Equation (1):

(1) $$\mathrm{DOE}=1-\frac{Integral\;area\;of\;olefinic\;protons\;after\;epoxidation}{Integral\;area\;of\;olefinic\;protons\;before\;epoxidation}\times 100$$

As shown in Table 1, the calculated *DOE* values of all epST-x samples are lower than the expected *DOE*, which may be due to the presence of inactive 1,2-vinyl parts and steric hindrance of the macromolecules during the epoxidation reaction.

**Figure 4:**
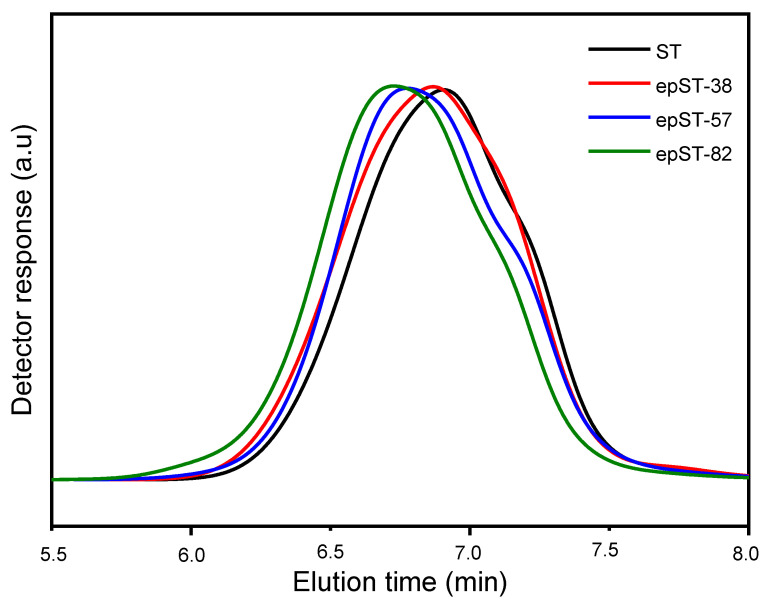
Gel Permeation Chromatography (GPC) plots of virgin ST and epST-x.

The GPC molar mass distribution curves obtained for the virgin ST and epST-x are shown in Figure 4. Only one prominent peak is present for each specimen with varying elution time. With increasing *DOE*, the chromatograms shift towards lower elution time, which is associated with changing molar mass. As shown in Table 2, the molar mass of the BCP gradually increases with increasing *DOE*, which is attributed to the introduction of oxygen atoms into the polymer main chain during the epoxidation reaction.

### 3.2. Morphology and Phase Behavior

**Figure 5:**
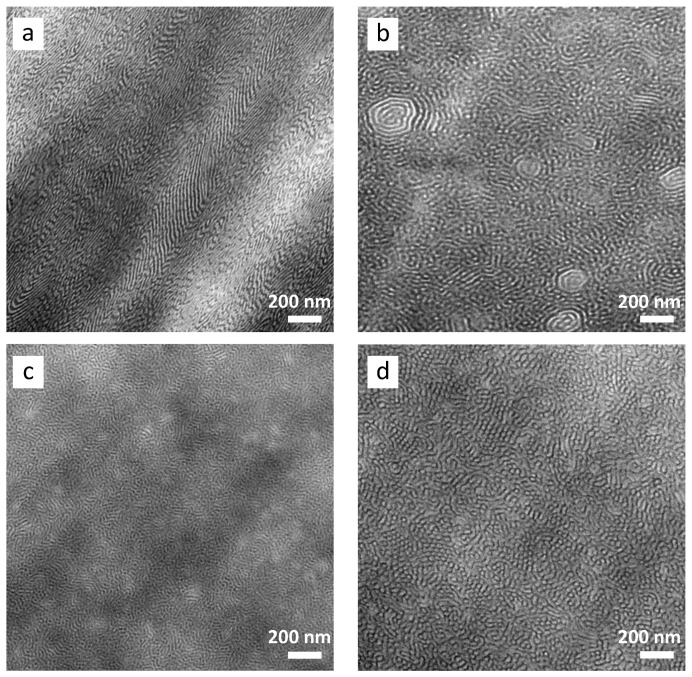
Transmission electron microscope (TEM) micrographs showing the morphology of virgin ST (**a**) and its chemically modified versions with varying epoxidation grades: epST-38 (**b**), epST-57 (**c**) and epST-82 (**d**).

The morphology of the films of ST and epST-x is shown in the TEM images in Figure 5. Following the classical phase diagram for linear SBS block copolymers, a hexagonal morphology is predicted at a total PS content of 75 wt% [41]. However, the TEM image of the virgin ST exhibit a complex morphology with the PB-rich phase (dark) and the PS-rich phase (bright) forming a mixture of curved, short and long lamellae-like structures of varying domain thicknesses (Figure 5a). Due to the presence of the random SB core in the molecules of ST, which will be considered as soft phase, the glassy domains are formed by the PS outer blocks, which comprise approximately 60 wt% of the copolymer. For such a PS:PB composition (hard soft ratio of 60:40) a lamellar morphology is actually expected. In addition, the highly asymmetric copolymer architecture can be responsible for shifting the boundaries between different micro phases [42].

The software Image J was used to measure the long period (*d*) of the structures and domain thickness of the PS-rich (*d_PS_*) and PB-rich phases (*d_PB_*) applying a Fast Fourier transformation (FFT) and a distance measuring tool. The FFT images (not shown here) of virgin ST show a significant orientation of the structure, but also a broad distribution of *d*. The long period of the oriented lamellae varies between 11 nm and 27 nm, with domain sizes *d_PS_* ranging from 9 nm to 12 nm and smaller PB domains *d_PB_* ranging from 4 nm to 7 nm. However, also distinctly widened lamellae with *d* of 35 nm to 45 nm could be observed, which is mainly attributed to the widening of *d_PS_* in the range of 14 nm and 20 nm.

**Figure 6:**
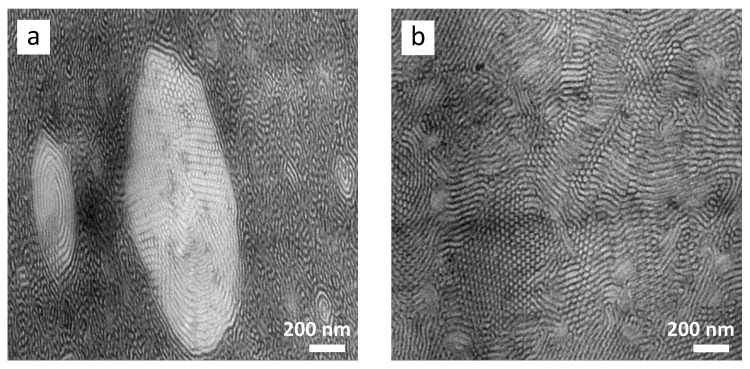
Local structural effects in the morphology of epST-38 (**a**) and epST-82 (**b**).

The epoxidation of ST causes an alteration in the BCP morphology, depending on the degree of epoxidation, as shown in Figure 5b–d. For sample epST-38, a mixture of short lamellae and cylinders were observed, with some lamellae forming circles, as demonstrated in Figure 5b. The FFT of the TEM images of sample epST-38 exhibited an orientation effect and a broad distribution of the long period, which is comparable to the virgin ST. Widened lamellae partly exhibit even larger d values up to 50 nm. However, a wide range of large circular areas with diameters of ~1 to 5 µm are spread over the whole sample, exhibiting a highly ordered cylindrical morphology as shown in Figure 6a. Assuming that the change in morphology is caused by the epoxidation of PB, these cylindrical domains are formed by PS and epoxidized PB.

Further increase in the epoxidation levels results in a distinct change in morphology as shown in Figure 5c. The chemically modified polymer epST-57 at intermediate epoxidation degree, exhibits a mainly cylindrical morphology mixed with disordered worm-like domains. The long period of the structure is distinctly reduced compared to sample epST-38 to *d* values between 15 nm and 32 nm. Furthermore, the structure exhibits marginal orientation. *d_PS_* is reduced to values mainly between 6 nm and 10 nm, whereas the PB domains are widened to values mainly between 8 nm and 10 nm, compared to epST-38 and ST. 

Increasing the *DOE* to 82 mol% leads to an increased amount of cylinders formed within the BCP structure of sample epST-82, while still exhibiting a wormlike morphology character (Figure 5d). Additionally, a distinct increase in the long period in the range of 20 nm to 36 nm and more distinct orientation could be observed compared to epST-57. The thickness of the PS-rich and PB-rich domain are distinctly increased to values of *d_PS_* ranging between 22 nm and 25 nm and *d_PB_* ranging between 10 nm and 15 nm, respectively. Interestingly, in few samples a highly ordered cylindrical structure could be observed (Figure 6b). However, this observation is limited to individual areas of the block copolymer, which could not be confirmed by the SAXS experiments as discussed below.

**Figure 7:**
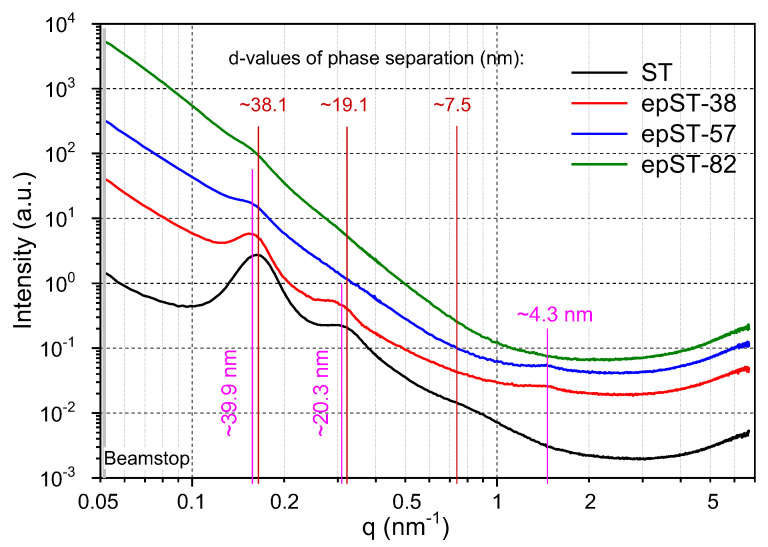
Small-angle X-ray scattering (SAXS) intensity profiles for virgin ST and its epoxidized grades at room temperature.

From the SAXS intensity profiles given in Figure 7, the lamellar morphology of the virgin ST could be confirmed. The long period *d* was found to be ~38.1 nm. However, concerning the broad and low number of reflections an indexing as cylinder morphology would be possible, too. Indeed, the weak reflections of higher orders pointing out a broad distribution of the domain sizes, which is reflected also in the TEM images.

For the epoxidized sample epST-38, a shift of the scattering peaks towards higher d-value of ~39.9 nm was found, which is in agreement with the observed long period range of the FFT images of this sample. The intensity of the reflection peaks significantly decreased compared to ST, indicating a lowering of a structure with long-range order. Sample epST-57 exhibits feeble reflections indicating a rather weak phase-separated structure with no significant order to long range, which confirms the observations from the TEM images, where a mainly wormlike structure was identified. For sample epST-82 the reflection peaks almost completely disappear, which indicates the absence of any ordered structure. This result is in contrast to the well-ordered morphology observed via TEM (Figure 6b) implying that the TEM imaging only represents a local structural phenomenon. It has to be noted, that the X-ray beam penetrates the whole cast film of about 1 mm thickness from top to bottom, whereas the electron beam to produce TEM images irradiates a cross section of the cast film of only ~60 nm, vertically to the X-ray beam. Consequently, the TEM images give information about a locally limited small area of the sample, whereas the X-ray scattering patterns represent an average morphology of the bulk material. Differences of morphology found by TEM and SAXS might have been caused by orientation effects that are generated during the sample preparation.

**Figure 8:**
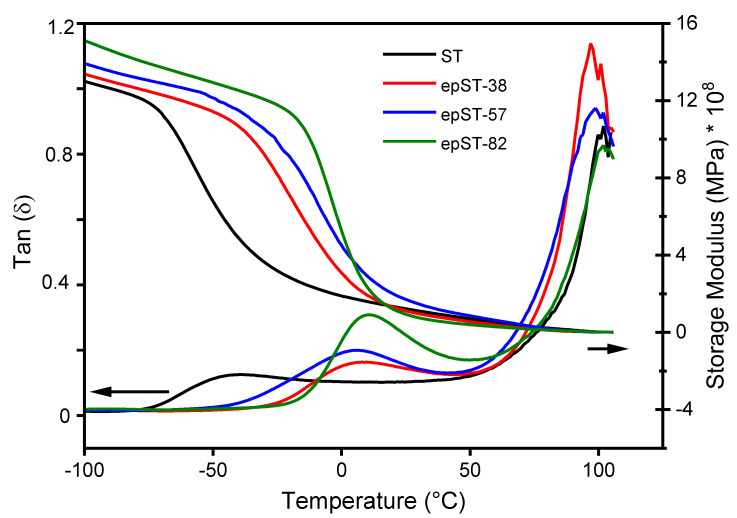
Loss factor *tanδ* (left) and storage modulus *G′* (right) versus temperature of virgin ST and epST-x.

Further information about the phase behavior of the investigated BCPs are provided by DMA. Damping coefficient (*tanδ*) and storage modulus (*G′*) of the polymers plotted as a function of temperature are given in Figure 8. The temperatures at the peak maximum of the *tanδ* curves were used to define the glass transition temperature (*T_g_*) of the material. Two distinct glass transition temperatures corresponding to two different phases could be identified for all the materials, one for the PB-rich phase and next for the PS-rich phase. For the homopolymers PB and PS, the *T_g_* are about 100 °C and −100 °C, respectively. For pure ST, the *T_g_* corresponding to the soft PS-*co*-PB phase was found to be at about −39 °C. Assuming complete miscibility of the PS phase and the PB phase in the PS-*co*-PB core the glass transition can be estimated using the Fox Equation (2) [43]:

(2) $$\frac{1}{T_{g}-soft}=\frac{\omega_{PS}}{T_{g}-PS}+\frac{\omega_{PB}}{T_{g}-PB}$$

with *ω_PS_* and *ω_PB_* representing the weight fractions of the pure PS and PB phases, respectively. The epoxidation of the PB chains leads to a distinct increase of *T_g-PB_* to ~8 °C, ~5 °C, and ~11 °C for epST-38, epST-57, and epST-82, respectively, which is also reflected in the *G′* curves as with increasing epoxidation grade the loss of *G′* shifts towards higher temperatures (Figure 8).

By the incorporation of epoxy groups, the PB chains of the copolymer become stiffer and lose their flexibility altering the hard-soft ratio in the PS-co-PB core, and therefore increasing the *T_g-PB_*. The glass transition of the PS-rich phase (*T_g-PS_*) was found to be 102 °C for virgin ST. The epoxidation of the block copolymer results in a slight shift of *T_g-PS_* towards lower temperatures of 97 °C for epST-38 and 99 °C for epST-57 and could be attributed to a slight decrease in phase separation as indicated by the quite worm-like disordered morphologies and weak reflection peaks. Interestingly, further increase of the epoxidation degree to 82 mol% again leads to a shift of *T_g-PS_* to a value of 102 °C. However, this might be due to an unstable molten state during the measurement at high temperatures causing an instrumental error.

### 3.3. Mechanical Behavior

**Figure 9:**
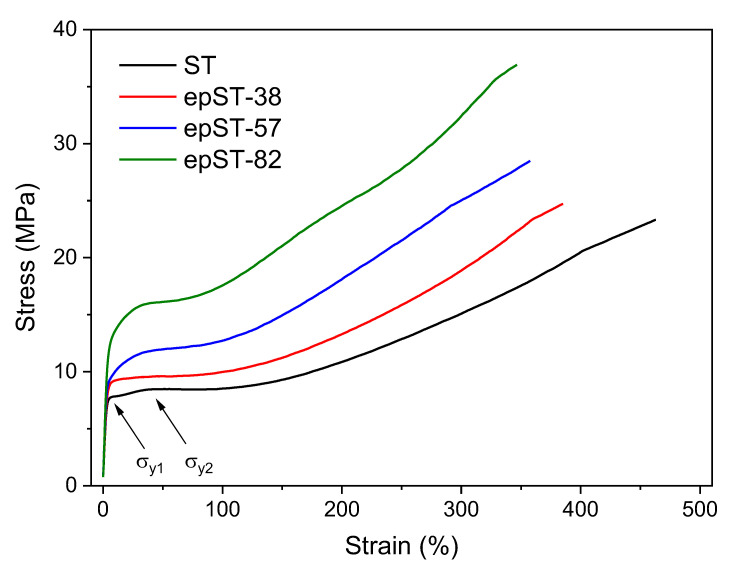
Tensile stress–strain diagrams of virgin ST and epST-x.

Tensile stress–strain curves of ST and epST-x are presented in Figure 9. Characteristic mechanical parameters are summarized in Table 3. The ST shows a pronounced ductile behavior with a large value of strain at break of 464%. Interestingly, this material exhibits two, but not very pronounced yield points at ~5% (*σ*_*y*1_) and ~36% (*σ*_*y*2_) of strain as marked in Figure 9.

This phenomenon of so-called “double-yielding” has been reported earlier [11] for similar star-shaped SB block copolymers and was also recently found in compression molded samples of the same material system [44]. With increasing degree of epoxidation, a clear increase of yield strength and a slight increase of the tensile modulus could be observed, indicating the enhanced stiffness of the material. The tensile strength increases from 23.5 MPa for ST up to 36.7 MPa for epST-82, whereas the strain at break values gradually decreases to 345%.

In summary, all the polymers show highly ductile behavior. The chemical modification indeed does not change the ductile nature of the polymer. The change in morphology from lamellar to more cylindrical and wormlike structures generally leads to a more elastomeric behavior due to a change in the deformation mechanism of the PS- and PB-domains, which compensates the stiffening of the material caused by epoxidation. The observed increase in strength of the sample at every level of strain for the chemically modified samples can be attributed to the molecular stiffening and reinforcement effect of the rubbery phase by the epoxide groups. Thus, the enhancement in strength and nominal reduction of ductility can be attributed to the increasing amount of epoxidized PB chains, which have, due to the structural change (incorporation of epoxy groups), higher stiffness and less flexibility than the non-treated PB. This effect was also reflected by the distinct increase of T*_g-PB_* of the epoxidized star block copolymers as discussed above.

## 4. Conclusions

The epoxidation of star shaped styrene-butadiene BCP comprising 75 wt% of polystyrene was carried out using *m*-CPBA and the structural and mechanical properties of the resulting materials were investigated. Following conclusions have been drawn:

- The experimental condition for epoxidation of BCP under precisely controlled conditions was optimized for the first time.
- Varying degrees of epoxidation (38 mol%, 58 mol%, and 82 mol%) were attained and validated by FTIR, ^1^H-NMR and GPC analyses.
- A transition in nanostructured morphology from a lamellae-like structure (in the BCP) to a cylindrical structure (for the sample with the highest epoxidation grade) could be observed via TEM, accompanied by a significant shift in soft phase *T_g_* values towards higher temperatures.
- The predominantly elastomeric property of the neat BCP was largely maintained while stiffness and tensile strength were enhanced by epoxidation.

Thus, the present study demonstrates the optimization of experimental conditions to meet the targeted degree of conversion and polarity. Hence, the epST shows the prospects of being used as reactive template for the fabrication of nanostructured thermosetting resins [34].

## Figures and Tables

**Table 1:** Degree of epoxidation (*DOE*) of ST and epST-x.

Sample Code	Targeted *DOE* (mol%)	Calculated *DOE* (mol%)
ST3	0	0
epST3-38	50	38
epST3-57	80	57
epST3-82	100	82

**Table 2:** Molecular characteristics from GPC of virgin ST and epST-x.

Sample Code	*M_n_* (g/mol)	*M_w_* (g/mol)	*Ð* = *M_w_*/*M_n_*
ST	82,000	172,500	2.1
epST-38	84,500	188,800	2.2
epST-57	88,300	201,200	2.3
epST-82	95,400	223,900	2.4

*M_n_* = Number average molar mass, *M_w_* = Weight average molar mass, *Ð* = Disparity.

**Table 3:** Some of the tensile mechanical characteristics of ST and epST-x.

Sample	*E_T_* (MPa)	*ε_B_* (%)	*σ_B_* (MPa)
ST3	182	464	23.5
epST3-38	187	386	24.7
epST3-57	211	360	28.5
epST3-82	221	345	36.7

*E_T_* = Tensile modulus, *ε_B_* = elongation at break, *σ_B_* = tensile strength.

## Data Availability

The data presented in this study are available within this article.
